# Draft Genome Sequence of an Indian Marine Cyanobacterial Strain with Fast Growth and High Polyglucan Content

**DOI:** 10.1128/genomeA.01334-17

**Published:** 2017-11-22

**Authors:** Ruchi Pathania, Ahmad Ahmad, Shireesh Srivastava

**Affiliations:** a Systems Biology for Biofuels Group, International Centre for Genetic Engineering and Biotechnology (ICGEB), New Delhi, India; b DBT-ICGEB Center for Advanced Bioenergy Research, International Centre for Genetic Engineering and Biotechnology (ICGEB), New Delhi, India

## Abstract

Marine cyanobacteria play an important role in global carbon cycling and are a potential source of polyglucans for biotechnological purposes. This report provides the draft sequence of an Indian marine cyanobacterium, *Synechococcus elongatus* BDU 130192, which shows fast growth and high polyglucan content. The genome sequence will help in understanding the unique properties of this organism.

## GENOME ANNOUNCEMENT

Cyanobacteria are important organisms ecologically and are being explored as biocatalysts to make a variety of molecules of interest. An important characteristic of cyanobacteria is to fix atmospheric carbon dioxide to various molecules of interest. Polyglucans, as polymers of glucose, are the major storage polymer in cyanobacteria. The polyglucan, and hence, glucose productivity of cyanobacteria are many fold greater than those of plant-based crops. The efficient synthesis of polyglucans by cyanobacteria makes them a potentially useful feedstock and a source of glucose for a variety of biotechnological purposes. Cyanobacteria also provide many other advantages, such as very simple nutrient requirement, very fast growth, easy transformability, etc. Additionally, the marine strains provide the advantage that they do not compete with freshwater resources. In this work, we present the draft genome sequence of an Indian isolate of *Synechococcus* species (designated *Synechococcus elongatus* BDU 130192) with fast growth and high polyglucan content. The strain was obtained from the National Facility for Marine Cyanobacteria (NFMC) at Bharathidasan University, India. In our hands, the growth of this strain is comparable to that of *Synechococcus* sp. strain PCC 7002 and polyglucan levels higher than those of *Synechococcus* sp. PCC 7002 (over 40% under normal culture conditions for the native strain compared to about 20% for *Synechococcus* sp. PCC 7002; our unpublished data).

Genomic DNA was isolated using the GenElute genomic DNA isolation kit (Sigma Aldrich). The concentration was measured spectrophotometrically, and the quality was analyzed using agarose gel electrophoresis. Paired-end libraries were prepared using the Agilent SureSelect adapters. The libraries were sequenced using the Illumina NextSeq 500 platform, yielding 150-bp paired-end raw reads. All the raw reads were quality checked for low-quality bases and adapter sequences using the NGS QC toolkit version 2.3.1 (1). The processed reads were assembled into contigs without any reference (*de novo*) using the Velvet-1.2.10 software (2). SSPACE_standard_3.0 (3) was used for scaffolding, and GapCloser-bin-v1-12-r6 (4) was used for gap closing. Gene prediction and annotation were done using the Rapid Annotation using Subsystems Technology (RAST) server (5).

The sequencing platform provided a total of 15,118,394 150-bp raw reads and 14,188,478 processed reads. About 95% of the processed reads were used for assembly. The genome of the bacteria was 3.2 Mb in size, about the size of a typical synechococcal species. This corresponded to 3,222 annotated genes. About 92.8% of the annotated proteins had some similarity with *Synechococcus* proteins, with 86% of the proteins (2,767 out of 3,222) having over 90% identity to *Synechococcus* proteins. Phylogenetic analyses identified that the organism had close similarity to *Synechococcus* sp. strain PCC 73109. The genome sequence and annotation will allow us to understand the capabilities of the organism and reveal the differences from other synechococcal strains. The information will also be useful for engineering the organism for biotechnological purposes.

### Accession number(s).

The sequences have been deposited at GenBank under accession number PCGE00000000. The version described in this paper is version PCGE01000000.
